# *Sarcocystis* Species Lethal for Domestic Pigeons

**DOI:** 10.3201/eid1603.090860

**Published:** 2010-03

**Authors:** Philipp Olias, Achim D. Gruber, Andrea Kohls, Hafez M. Hafez, Alfred Otto Heydorn, Heinz Mehlhorn, Michael Lierz

**Affiliations:** Freie Universität Berlin, Berlin, Germany (P. Olias, A.D. Gruber, A. Kohls, H.M. Hafez, A.O. Heydorn, M. Lierz); Heinrich-Heine-Universität, Düsseldorf, Germany (H. Mehlhorn); 1 Current affiliation: Justus-Liebig-Universität Giessen, Giessen, Germany.

**Keywords:** Apicomplexa, protozoa, polyuria, depression, torticollis, encephalitis, ribosomal, RNA, 18S, 28S, parasites, dispatch

## Abstract

A large number of *Sarcocystis* spp. infect birds as intermediate hosts, but pigeons are rarely affected. We identified a novel *Sarcocystis* sp. that causes lethal neurologic disease in domestic pigeons in Germany. Experimental infections indicated transmission by northern goshawks, and sequence analyses indicated transnational distribution. Worldwide spread is possible.

A large number of *Sarcocystis* spp. (Protozoa: Apicomplexa) may infect birds as intermediate hosts, but wild Columbiformes, which include pigeons, are rarely affected (*1*–*3*). Among the few species affecting domestic poultry are *S. horvathi* and *S. wenzeli,* which affect chickens, and *S. rileyi*, for which ducks are intermediate hosts (*4*,*5*). *S. falcatula* has been known to cause clinical disease in pigeons only after experimental infection; whether this species is pathogenic under natural conditions is not known (*6*).

We recently reported an emerging neurologic disease with lethal outcome for domestic pigeons (*Columba livia* f. *domestica*) in Berlin, Germany, caused by a novel *Sarcocystis* sp. (*3*). When compared with *S. falcatula* and other bird-infecting *Sarcocystis* spp. such as *S. lindsayi*, the novel species differed in its ultrastructural and genetic features (*3*,*6*,*7*). Clinical signs in naturally infected pigeons, which were similar to those caused by *Paramyxovirus*-1 or *Salmonella typhimurium* var. cop. infection, were depression, polyuria, torticollis, opisthotonus, paralysis, trembling, and death. Pigeons had numerous parasitic cysts in their muscles. We hypothesized that pigeons serve as intermediate hosts in a 2-host life cycle characteristic for *Sarcocystis* spp., in which pigeons are infected by ingestion of sporocysts shed in feces from an unidentified definitive host (*8*). We further characterized the parasite genetically, identified its definitive host and life cycle, and determined its causative role in this novel disease of pigeons.

## The Study

**Figure 1:**
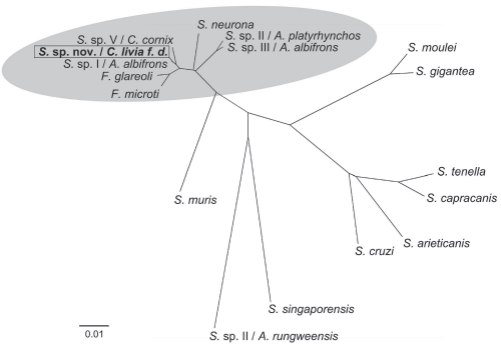
Phylogenetic comparison of novel *Sarcocystis* sp. with related *Sarcocystis* spp. Tree constructed by neighbor-joining using Kimura 2-parameter method based on the partial 18S rRNA gene comprising 1,391 bp and the D2 region of 28S rRNA gene comprising 325 bp of the novel *Sarcocytis* sp. (GenBank accession no. GQ245670/FJ232949) and the following available *Sarcocystis* sequences: *Frenkelia microti* (*S. buteonis*) (AF009244/AF044252); *Frenkelia glareoli* (*S. glareoli*) (AF009245/AF044251); *S*. sp. (cyst type I) ex *Anser albifrons* (EU502869/EF079886); *S*. sp. (cyst type V) ex *Corvus cornix* (EU553478/EF079884); *S. neurona* (U07812/AF092927); *S*. sp. (cyst type II) ex *Anas platyrhynchos* (EU553477/EF079887); *S*. sp. (cyst type III) ex *A. albifrons* (EU502868/EF079885); *S. moulei* (L76473/AF012884); *S. gigantea* (L24384/U85706); *S. tenella* (L24383/AF076899); *S. capracanis* (L76472/AF012885); *S. arieticanis* (L24382/AF076904); *S. cruzi* (AF017120/AF076903); *S. singaporensis* (AF434054/AF237617); *S*. sp. II ex *Atheris rungweensis* (AF513490/AF513493); *S. muris* (M64244/AF012883). Highlighted area indicates branch of bird-infecting *Sarcocystis* spp. Scale bar indicates genetic distance.

In 2008, DNA was extracted from the pectoral muscles of a pigeon that had been naturally infected during a recent outbreak in Germany. DNA sequences encoding the 18S rRNA and D2-region of the 28S rRNA of the *Sarcocystis* sp. were PCR amplified and sequenced, after which multiple sequence alignments and construction of phylogenetic relationships were conducted (*3*,*9*–*11*). The 18S rRNA and D2-region sequences were deposited in the GenBank database (accession no. GQ245670). Comparison of the 18S rRNA with published sequences of *Sarcocystis* spp. identified 1 matching sequence of 783 bp isolated from a Cooper’s hawk (*Accipiter cooperii*) (GenBank accession no. EU810398). Sequence analysis of a combination of the 18S rRNA and D2 region showed close homologies to other bird-infecting *Sarcocystis* spp. (Figure 1) and only 4 nt differences from a *Sarcocystis* sp. found in a white-fronted goose (*Anser albifrons*) (*12*).

To identify the definitive host, we conducted an experimental infection study using predators that had possible contact with the naturally infected pigeons: 2 dogs (*Canis familaris*, beagles), 2 ferrets (*Mustela putorius furo*), 2 rats (*Rattus norvegicus* f. *domestica*), 2 mice (*Mus musculus domesticus*), 2 northern goshawks (*Accipiter gentilis*), and 2 Gyr-Saker hybrid falcons (*Falco rusticolus* × *Falco cherrug*). Fecal samples from all animals were negative for parasites before infection. Each animal was fed 1 regular-sized meal of pectoral muscle of 2 racing pigeons naturally infected with cysts from the 2008 outbreak in Germany (*3*). Starting on day 6 after infection, only the goshawks shed sporocysts (7.9 × 11.9 µm) in their feces. Microscopically, many oocysts (each containing 2 sporocysts) were detected in the mucosa of the small intestine, which is characteristic for *Sarcocystis* spp. Identical D2-region sequences were detected in sporocysts from goshawk feces and in *Sarcocystis*-infested muscles from naturally infected pigeons. All other animals failed to shed sporocysts. No clinical signs developed in the goshawks or the other animal species.

To experimentally reproduce the disease, we infected domestic pigeons with an oral dose of purified sporocysts from 1 goshawk. Pectoral muscle biopsy samples taken before experimental infections were free of parasites. Fecal examination confirmed absence of *Salmonella* spp. and endoparasites. We separated 16 pigeons into 8 groups of 2 birds each and gave pigeons in groups 1–7 infectious doses (IDs) of 3 × 10^6^, 3 × 10^5^, 10^5^, 8 × 10^4^, 10^4^ , 10^3^, or 10^2^. Pigeons in group 8 served as controls. Animals with neurologic signs were euthanized, and surviving pigeons were euthanized at 59 and 120 days after infection, respectively.

Pigeons in groups 1–4 (IDs 3 × 10^6^ to 8 × 10^4^) died within 12 days after infection. After 8 weeks of infection, severe and moderate neurologic signs developed in pigeons of groups 5 (ID 10^4^) and group 6 (ID 10^3^), respectively. After 9 weeks of infection, pigeons in group 7 (ID 10^2^) had mild to moderate neurologic signs. Control pigeons of group 8 remained free of clinical signs throughout the study.

**Figure 2:**
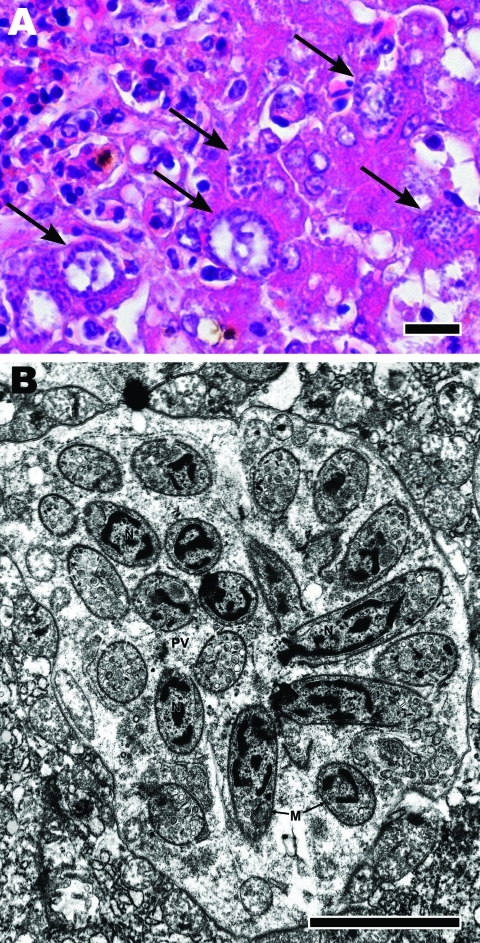
A) Microscopic appearance of liver with tissue necrosis, lymphohistiocytic inflammation, and *Sarcocystis* schizonts (arrows) in a pigeon 8 days after infection with 10^5^ *Sarcocystis* sporocysts. Hematoxylin and eosin stain; scale bar = 20 μm. B) Transmission electron micrograph of a hepatocyte from liver in panel A, containing a schizont, forming cross-sectioned and longitudinally sectioned merozoites. N, nucleus; PV, parasitophorous vacuole; M, merozoite. Scale bar = 20 μm.

Histologic examination of livers from pigeons in groups 1–4 showed multifocal severe necroses with numerous parasitic stages (Figure 2, panel A). Pigeons in groups 5–7 had marked encephalitis, myositis, and *Sarcocystis* cysts in skeletal muscles (pectoral, gastrocnemius, and neck) but not in the brain. Control pigeons had no microscopic lesions in any organs. Neither *Salmonella* spp. nor a hemagglutinating agent was cultured from any pigeon.

Electron microscopic examination of livers was performed as previously described (*3*). Parasitic stages, identified as developmental stages of schizonts, were seen in livers of pigeons of groups 1–4 (Figure 2, panel B). Simultaneous development of merozoites above a giant nucleus of the schizont, the typical endopolygeny for a *Sarcocystis* parasite, was noted. Identical D2-region DNA sequences were detected in the livers and skeletal muscles from all experimentally infected pigeons and from the *Sarcocystis*-infested muscles from naturally infected pigeons.

## Conclusions

This study identifies the northern goshawk as the probable definitive host of a recently described novel *Sarcocystis* sp. in domestic pigeons in Germany, indicating a typical prey–predator transmission cycle (*3*). The clinical signs and organ lesions of experimentally infected animals mirror those of naturally infected racing pigeons.

Previous results suggested that this parasite represents a new *Sarcocystis* sp. and is genetically distinct from *S. falcatula* (*3*). Our further sequence analyses indicated that the novel *Sarcocystis* sp. is closely related or even identical to a *Sarcocystis* sp. previously detected in a Cooper’s hawk in the state of Georgia, USA (*13*). Cooper’s hawks are widespread in North America and in some areas hunt mainly pigeons (*14*). Further phylogenetic analyses showed that this *Sarcocystis* sp. is closely related but distinct from other bird-infecting *Sarcocystis* spp. (Figure 1).

Goshawks are widely distributed in the Northern Hemisphere, where the domestic pigeon is also common. Throughout Europe, pigeons are the principal prey for goshawks (*15*). Thus, we speculate that this *Sarcocystis* sp. may be present in other countries or could easily be introduced and become endemic elsewhere. It remains to be shown whether other avian species, in addition to pigeons, may serve as intermediate hosts. This assumption is supported by a close sequence homology between this *Sarcocystis* sp. and a *Sarcocystis* sp. previously found in striated muscles of a white-fronted goose (Figure 1).

Among the experimentally infected pigeons, different diseases were caused by different infectious doses. Pigeons infected with high doses died 7–12 days after infection and had massive parasite-induced liver necroses; those infected with lower doses had central nervous signs, which did not develop until 8 weeks after infection. The late occurrence of brain lesions and the absence of parasitic stages from the brain suggest an indirect, currently unknown, mechanism of encephalitis that awaits further clarification.

In conclusion, the emerging *Sarcocystis* sp. cycles between northern goshawks and domestic pigeons and is highly pathogenic for the pigeons after they ingest low doses of sporocysts. Pigeon sport and falconry should therefore be considered as risk factors for further disease transmission.
